# Agglomeration Efficacies of Simple Salts on Charged Gold Nanocrystals with Mixed Ligand Shells: A High-Throughput Study

**DOI:** 10.1021/acsmaterialsau.6c00051

**Published:** 2026-05-09

**Authors:** Albenc Nexha, Bart-Jan Niebuur, Simon Blum, Tobias Kraus

**Affiliations:** † 28391 INM-Leibniz Institute for New Materials, Campus D2 2, Saarbrücken 66123, Germany; ‡ Saarland University, Colloid and Interface Chemistry, Saarbrücken 66123, Germany

**Keywords:** mixed thiol coated gold nanocrystals, Hofmeister series, colloidal stability, micropippeting robot, high throughput protocol, machine learning

## Abstract

Salts induce the agglomeration and assembly of gold nanocrystals that are stabilized by charged ligand shells. This destabilization is known to partially deviate from the predictions of classical DLVO theory for larger colloids, but existing studies focus on limited concentration ranges or ion types. Here, we use a high-throughput approach to test the agglomeration efficacy of 17 different salts at concentrations ranging from 0.16 mM to 2 M on negatively charged nanocrystals with shells of 11-mercaptoundecanoic acid and/or triethylene glycol mono-11-mercaptoundecyl ether. Automated pipetting is used to create a large dataset of close to 10000 UV–vis absorbance spectra. We analyze the spectral shifts to find the onset of agglomeration, identify critical salt concentrations, and characterize the nature of the agglomeration transition. The results are compared to classical DLVO theory using conventional analysis, and the effects of ion concentration and anion valency are shown to be consistent with DLVO predictions. Cation valencies only partially follow the predictions, suggesting local ion-specific interactions that dominate when screening lengths reach molecular length scales. A Random Forest Regression model is used as additional “black box” analysis of the results to correlate the ionic strength, ligand shell composition, and intrinsic ion properties, and to rank their relative importance to the colloidal stability of gold nanocrystals. The ranking combines classical DLVO effects and specific ion interactions with subtle effects on the agglomerate structure that affect plasmon resonance shifts, providing a complementary interpretation of the data.

## Graphical abstract

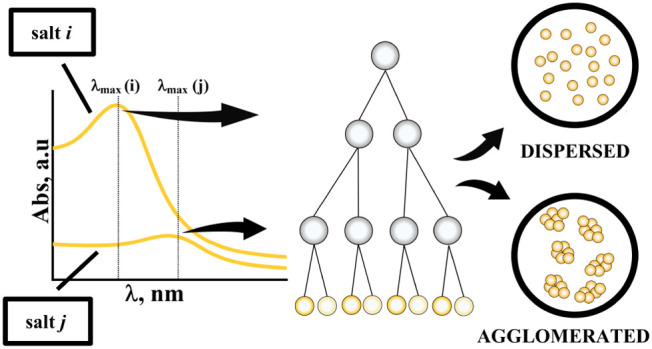

## 1 Introduction

Colloidal gold nanocrystals (Au NCs) have optical properties that depend on size, shape, crystal structure, and chemical surface states.^1,2^ They are applied in fields ranging from photonic metamaterials,^3^ printed electronics,^4^ medical assays,^5,6^ to therapeutics,^7^ where their low toxicity is exploited. The position of the localized surface plasmon resonance (LSPR) peak has been used in sensing, e.g., to detect *Staphylococcal enterotoxin A* with a strong optical response visible to the naked eye.^6^ Rod-like Au NCs with LSPRs in the infrared for photothermal therapy,^7^ are already in clinical trials (ClinicalTrials.gov, ID: NCT02680535).

Agglomeration and assembly affect the optical and chemical properties of Au NCs. Their colloidal stability depends on the balance between attractive van der Waals (vdW) interactions of the metallic cores and repulsive interactions (electrostatic, steric, or entropic).^8,9^ Agglomeration can also be caused by specific interactions, e.g., mediated by DNA attached to the Au NC surfaces,^10,11^ a topic that we do not cover here. Agglomeration yields disordered particle arrangements that often range between reaction- and diffusion-limited structures (RLA/DLA),^12–14^ while assembly can yield ordered arrangements, for example, “*superlattices*”, “*supercrystals*”, or “*supraparticles*”.^15–17^ Whether order or disorder emerges depends on the interaction potential between the particles and their trajectory in the free energy landscape.^18^ This landscape may change with time, e.g., when dispersed Au NCs are slowly destabilized by the addition of salt.^18^

The colloidal stability of Au NCs is routinely tuned using charged species adsorbed on the surface, such as negatively charged citrates,^19^ halides,^20^ positively charged cetyltrimethylammonium bromide (CTAB),^21^ or polyethylenimine (PEI).^22^ While these are already adsorbed during synthesis, organic thiols may be adsorbed in postsynthesis modification to adjust the charge.^23–25^ Thiols adsorb rapidly, forming an Au–S covalent bond (184 kJ/mol) and can densely coat the gold cores in self-assembled monolayers (SAMs).^26,27^

Classic DLVO theory predicts that the concentration and valence of ions in the electrolyte affect colloidal stability. As the ionic strength increases, the Debye screening length decreases, thereby reducing the energy barrier for aggregation.^28^ The basic theory does not account for size-dependent surface curvature or discrete ion effects.^29^ It is known to underestimate the stability of nanocrystals in high-salinity media, where non-DLVO forces such as hydration or steric repulsions from adsorbed ligands become dominant.^29^ DLVO theory assumes inert particle surfaces, but Au NCs are catalysts that may undergo structural or surface changes.^30^ Consequently, current research in colloidal science aims to extend and amend DLVO theory with simulations and experiments.^31–33^

Current research tested whether existing non-DLVO concepts for the stability of large molecules can be applied to Au NCs.^34–38^ For example, the Hofmeister series ranks the effect of salts on the solubility of proteins and defines strongly and weakly hydrated ions.^31^ The strongly hydrated ions stabilize the proteins, reduce their solubility, and eventually cause precipitation, while the weakly hydrated ions increase solubility.^31^ Recent work evaluated whether these principles can be applied to the stability of Au NCs, too.^34–38^ Christau et al. tested the effect of different alkali halide ions on dispersions of Au NCs coated with poly­(*N*-isopropylacrylamide) (PNIPAM).^34^ The salts induced aggregation with efficiencies of NaF < NaBr < NaCl < KCl, without an obvious relation to ion properties of the Hofmeister series. Other researchers quantified the colloidal stability of Au NCs with 11-mercaptoundecanoic acid shells in aqueous solutions of MCl (M = Li^+^, Na^+^, K^+^, Rb^+^, Cs^+^).^38^ Their aggregation efficiency followed the order CsCl > KCl > LiCl > NaCl > RbCl.^38^ This order does not correlate with the size of hydrated cations M^+^ and does not follow the Hofmeister series.^38^ The three anions Cl^–^, NO_3_^–^, and SO_4_^2–^ led to identical aggregation efficiencies for M = Na^+^.^38^ Experiments with thiol-coated Au NCs showed that divalent ions (Mg^2+^, Ca^2+^) induced aggregation at lower concentrations than monovalent ions (Na^+^, K^+^).^39^ Finally, Ritzert et al. reported that polarizable anions such as I^–^ and SCN^–^ caused chemical reactions in dispersions of covalently bound mercaptopropionic acid on Au NCs: I^–^ promoted particle fusion and rapid loss of the nanoparticle structure, while SCN^–^ induced aggregation but preserved the nanoparticle structure longer before sedimentation.^35^

It remains challenging to identify which effects, beyond those considered by DLVO, are relevant in colloidal nanoparticle stability. There is no closed extension of DLVO available at present that would cover all relevant effects. Gradual or partial agglomeration is difficult to quantify, and it is not trivial to define suitable endpoints in order, e.g., to classify “*stable*” versus “*unstable*” dispersions as a function of ion concentration. If no theory is available to guide the choice of concentrations and if the endpoints are hard to define, numerous experiments are required to avoid misinterpretation. The currently employed experimental techniques are comparatively time-consuming and do not scale well to hundreds or thousands of combinations and their repeats. Finally, differences in agglomerates and the emergence of ordered assembly structures are detected through electron microscopy or X-ray scattering today, methods that are even slower than the sample preparation using conventional methods.

Recent progress in high-throughput experimentation and the emergence of powerful black-box models (based on “machine learning” or, more generally speaking, exploratory data analysis) can guide the way toward overcoming these limitations.^40–42^ Here, we use an agnostic approach that does not make assumptions about the underlying mechanisms of agglomeration and assembly of Au NCs. We combined parallel robotic pipetting, microplates, and automated optical analysis to induce and analyze agglomeration. While the choice of ions was inspired by previous work on the Hofmeister series, we did not make any assumptions based on it and tested a wide concentration range. The Au NCs’ charge was independently varied using two different thiols for the modification of their surfaces. Similar automated processes have already been applied to discover new catalysts,^40–42^ and in the scale-up of new organic molecules,^43^ but not for the analysis of the colloidal stability of Au NCs, to our knowledge.

We combined high-throughput experimentation with Random Forest Regression, an ensemble learning algorithm that is scalable and yields results that can be compared to classical interpretation.^44^ The Au NCs that we used here carried self-assembled monolayers (SAMs) of organic thiolates with negative or neutral ω-groups. We selected them because these molecules are relatively strongly adsorbed, with comparatively low concentrations of free ligands in solution. The free ligands were clearly different from the ions used in the agglomeration studies, avoiding interference. In contrast, citrates^34,35^ are weakly adsorbed and easily removed in washing steps. Polymers^45^ can cause additional depletion interactions when released into the electrolyte. Finally, the SAMs protect the gold surface to some degree and prevent ions from etching the AuNPs.^34,35^

We used a micropipetting robot linked to a microplate absorbance reader to extract the wavelengths of the LSPR absorbance peak of Au NCs for a fast screening of colloidal stability. The nanocrystals were water-soluble and functionalized with a mixture of 11-mercaptoundecanoic acid (hereafter MUA), a negatively charged ligand, and triethylene glycol mono-11-mercaptoundecyl ether (hereafter TEG), a neutral ligand, to tune the surface charge. The thiol-coated Au NCs were then exposed to different salts and subsequently read optically via a microplate. We analyzed the change in optical properties of the dispersions as a function of the ratio of MUA:TEG ligands (only MUA, excess of MUA, and an equimolar ratio), the nature of the ions in the salts (strongly and weakly hydrated), their radii (ranging from 60 to 240 pm), and valency (1, 2, or 3), as well as their concentrations (0.00016 to 2 M). The data generated were analyzed by a machine learning algorithm, where we used the plasmonic peak shift as an indicator of Au NCs agglomeration.

## 2 Materials and Methods

### 2.1 Materials

Tetrachloroauric (III) acid trihydrate (≥99% trace metal basis), trisodium citrate dihydrate (≥99%), citric acid monohydrate (≥99%), potassium carbonate (≥99%), and tannic acid (MW = 1701 g/mol) for particle synthesis were purchased from Sigma-Aldrich. The ligands, 11-mercaptoundecanoic acid (95%, MUA) and triethylene glycol mono-11-mercaptoundecyl ether (TEG), and all salts were purchased from Sigma-Aldrich.

### 2.2 Synthesis of Gold Nanocrystals

The citrate-coated Au NCs were synthesized using seeded growth,^19^ following a slightly adapted protocol. Briefly, within a 250 mL three-neck flask, a mixture containing aqueous solutions of sodium citrate (150 mL, 2.2 mM), tannic acid (0.1 mL, 2.5 mM), and potassium carbonate (1 mL, 150 mM) was added. The mixture was heated using an oil bath. When the temperature reached 70 °C, an aqueous HAuCl_3_ solution (1 mL, 25 mM) was swiftly injected. The color of the solution changed rapidly from black to red within 2 min. The mixture was allowed to react at this temperature for an additional 5 min to ensure complete reaction. These resulting Au NCs (3.5 nm in diameter) were immediately used as seeds.^19^ First, 55 mL of the prepared seed solution was diluted with 55 mL of 2.2 mM sodium citrate in water in the same three-neck flask and heated. Second, at 70 °C, two volumes of aqueous HAuCl_3_ (each 0.5 mL, 25 mM) were injected at a time interval of 10 min. These two steps were repeated 6 and 12 times, respectively, until the Au NCs reached a size of approximately 12 nm, as previously reported.^19^ The resulting Au NCs dispersion had a concentration of 1 mg/mL, and the particles were covered with citrate ions.

### 2.3 Ligand Exchange

Solutions of the thiols MUA and TEG at different molar ratios (2.4 mM MUA; 2.4 mM MUA and 1.2 mM TEG; and 2.4 mM MUA and 2.4 mM TEG) were added to the dispersions of 12 nm Au NCs.^46,47^ Equimolar ratios of NaOH were added to aid the dissolution of MUA in distilled water. The dispersions were stirred overnight at room temperature. The resulting Au NCs with MUA or mixed ligand shells were concentrated to 2 mL by repeated centrifugation at 10500 rpm for 1 h and then washed twice with Millipore water, as previously reported.^46,47^

### 2.4 Characterization of the Nanocrystals

The size and shape of the citrate-coated Au NCs were determined from TEM micrographs recorded on a JEOL JEM 1010 microscope. An aqueous dispersion of the citrate-coated Au NCs (approximately 15 μL) was drop-cast onto a carbon-coated grid and allowed to dry at room temperature for approximately 24 h. The size distribution of the particles was measured using the ImageJ software by accounting approximately 100 individual nanoparticles. We measured Small-Angle X-ray Scattering (SAXS) for a statistically more relevant analysis of the shape and size of the citrate-coated Au NCs. SAXS patterns were recorded using a Xeuss 2.0 laboratory setup (Xenocs SAS, Grenoble, France). The incident beam from a copper K_α_ source with a wavelength of λ = 0.154 nm was collimated and focused on the sample with a spot size of 0.25 × 0.25 mm^2^. Two-dimensional scattering patterns were recorded by a Dectris Pilatus 3R 1M detector with an acquisition time of 3600 s and a sample-to-detector distance of 1214 mm, as calibrated using a silver behenate standard. The scattering patterns were azimuthally averaged to obtain the scattering intensity, *I*, depending on momentum transfer q = 4πλ^–1^sin *θ*, with *θ* being the scattering angle. UV–visible spectra were acquired with a Cary (USA) 300 UV–vis spectrometer within the wavelength range of 400 to 800 nm, using a quartz cuvette filled with a 1 mg/mL dispersion of gold nanocrystals in water. The average hydrodynamic diameter of the nanoparticles was determined from Dynamic Light Scattering (DLS) using an Anton Paar Litesizer 500. A single-use poly­(methyl methacrylate) cuvette was filled with water-soluble gold nanoparticles. A semiconductor laser diode emitting at 658 nm with a power of 40 mW was used as the light source. A log-normal function was used to extract the hydrodynamic diameter of the nanoparticles. The ratios of the adsorbed ligand quantities were analyzed using proton nuclear magnetic resonance (^1^H NMR). ^1^H NMR data of digested samples (with the gold cores removed to avoid peak broadening) were acquired on a Bruker Avance 300 spectrometer using D_2_O as a reference solvent with 512 scans, a relaxation delay of 10 s, and an operating temperature of 298 K. The gold nanoparticles were prepared for ^1^H NMR by overnight digestion in 20 μL aqua regia followed by dilution with 500 μL D_2_O. The spectra were postprocessed using the TopSpin software. The zeta potentials of the thiol-coated nanoparticles were measured using a Nano ZS90 (Malvern, USA) Zetasizer instrument.

### 2.5 Automated Agglomeration Studies

An Integra Assist Plus micropipetting robot equipped with an Integra Voyager 8-channel pipette from INTEGRA Biosciences AG was set to prepare mixtures of Au NC dispersions and salt solutions. It was controlled using the Vialab software,^48^ with an algorithm that allows one to create automated pipetting protocols (the algorithm is documented in the SI). We used single-use polysterene 384-well plates from Corning. A total of 17 different salts (summarized in Table 1) at 48 different concentrations were tested for each of the 3 Au NC shell types. Each sample was prepared in quadruplicate, so that 9792 measurements were recorded in total. Stock concentrations for each salt were prepared and then diluted to create final salt concentrations between 0.16 mM and 2 M in the dispersions. Each automated pipetting run to create the desired electrolytes and mix them with Au NCs required approximately 40 min, with Au NCs added during the last 5 min of the process. The well plates were then moved to a Cary UV–vis spectrometer (typical transport duration: 5 min) equipped with a Spark Multimode Microplate Reader from Tecan Trading AG, which is equipped with a xenon arc discharge lamp. The absorbance was recorded within the wavelength range of 450 to 750 nm, using a step size of 6 nm, and completed within a total of 30 min, with an integration time of 5 s for each well. This limited resolution reduced the delay in readout and thus differences in the age between different samples. As a reference, 24 spectra of deionized water were recorded under identical conditions, averaged, and subtracted from the data.

**Table 1:** Overview of All Investigated Salt Types and Their Valency, Cation Radius *R* ^+^, Anion Radius *R* ^–^, Cation Polarizability *α* ^+^, and Anion Polarizability *α* ^–^ of Their Respective Ions

Salt type	Cation valency	Anion valency	*R* ^+^ [pm]/^ref^	*R* ^–^ [pm]/^ref^	α^+^ [Å^3^]/^ref^	α^–^ [Å^3^]/^ref^
AlCl_3_	3	1	50/^49^	180/^49^	0.038/^50^	3.5/^51^
CaCl_2_	2	1	103/^49^	180/^49^	0.44/^51^	3.5/^51^
FeCl_2_	2	1	72/^49^	180/^49^	0.589/^50^	3.5/^51^
GuaCl	1	1	220/^52^	180/^49^	4.44/^52^	3.5/^51^
KCl	1	1	141/^49^	180/^49^	0.81/^51^	3.5/^51^
MgCl_2_	2	1	70/^49^	180/^49^	0.08/^51^	3.5/^51^
NH_4_Cl	1	1	136/^53^	180/^49^	2.0/^54^	3.5/^51^
SrCl_2_	2	1	125/^49^	180/^49^	0.81/^51^	3.5/^51^
NaCl	1	1	97/^49^	180/^49^	0.18/^51^	3.5/^51^
Na_2_CO_3_	1	2	97/^49^	189/^53^	0.18/^51^	5.39/^55^
Na_2_HPO_4_	1	2	97/^49^	238/^49^	0.18/^51^	6.84/^56^
Na_2_S_2_O_3_	1	2	97/^49^	251/^53^	0.18/^51^	8.29/^57^
Na_2_SO_4_	1	2	97/^49^	242/^49^	0.18/^51^	4.43/^58^
NaBr	1	1	97/^49^	198/^49^	0.18/^51^	4.6/^51^
NaF	1	1	97/^49^	124/^49^	0.18/^51^	1.3/^51^
NaH_2_PO_4_	1	1	97/^49^	238/^49^	0.18/^51^	5.79/^59^
NaI	1	1	97/^49^	225/^49^	0.18/^51^	7.5/^51^

### 2.6 Data Analysis

The measurements resulted in 9792 optical absorption datasets. To account for their limited resolution, we determined the wavelength of maximum absorption λ_LSPR_ using interpolation. This wavelength corresponds to the position of the localized surface plasmon resonance (LSPR) frequency. Piecewise cubic polynomials were fitted between points around the LSPR peak in each dataset’s maximum using the SciPy package in Python. Scalar minimization, following the Brent method and also performed using the SciPy package, was used to find the wavelength at the maximum of the interpolated function.

We employed machine learning algorithms for the automated interpretation of the data (Zenodo link: DOI: 10.5281/zenodo.19666143). Four commonly used ensemble algorithms were selected for their robustness to noise and capability of capturing nonlinear relationships between descriptors and target variables, namely, Gradient Boost Regression (GBR),^60^ XGBoost Regression (XGBR),^61^ CatBoost Regression (CBR),^62^ and Random Forest Regression (RFR).^63^ They were implemented in Python using existing libraries (sklearn for GBR and RFR, xgboost for XGBR, and catboost for CBR) and tested for their performance using the metrics “coefficient of determination” (*R* ^2^), “root-mean-square error” (RMSE), and “mean absolute error” (MAE). Using an 80/20 train-test split of the recorded data, RFR scored best across these performance metrics (Table S1, SI) and was chosen for further interpretation of the data. This algorithm uses ensembles of decision trees (regression trees) to model the relationship between descriptors and a continuous target variable. A decision tree recursively splits the data into subsets based on descriptor values so that the target variable is as similar as possible within each subset. Repeated splitting progressively reduces the variance of the target values within these subsets, improving the tree’s ability to predict the target variable from the descriptors. By training many decision trees on different subsamples of the data and averaging their predictions, RFR reduces overfitting and achieves higher predictive accuracy than a single decision tree. An RFR model consisting of 25 decision trees was found optimal; increasing the number further did not improve the coefficient of determination, *R* ^2^.

## 3 Results and Discussion

### 3.1 Synthesis, Characterization, and Ligand Exchange of Gold Nanocrystals

Aqueous dispersions of Au NCs coated with citrate ions were synthesized via a seeded growth protocol.^19^ Transmission electron micrographs (TEM) of the nanoparticles revealed spheres with an average diameter of 11.7 ± 1.2 nm (Figure 1A). High-resolution TEM images indicated lattice fringes of approximately 0.22 nm (Figure S1, SI), matching with [111] crystal planes in a face-centered cubic (FCC) gold crystal lattice. A hydrodynamic diameter of 11.4 nm was deduced from Dynamic Light Scattering (DLS) (Figure 1B). The Small-Angle X-ray Scattering (SAXS) pattern had a shoulder at ∼0.5 Å^–1^ and a series of intensity oscillations at larger *q*-values, indicative of dispersed spherical nanocrystals with a narrow size distribution (Figure 1C). A model fit of the data, using a form factor of spheres with a Gaussian size distribution,^64^ indicated an average diameter of 11.4 nm with a standard deviation of 0.12 nm. UV–vis absorbance spectra revealed a clear LSPR peak (λ_LPSR_) at 522 nm (Figure 1D), as expected for well-dispersed Au NCs in a diameter range of approximately 15 to 20 nm.^65^ Overall, the results extracted from TEM, DLS, SAXS, and UV–vis are in close agreement with each other, confirming the successful formation of gold nanocrystals.

**Figure 1:**
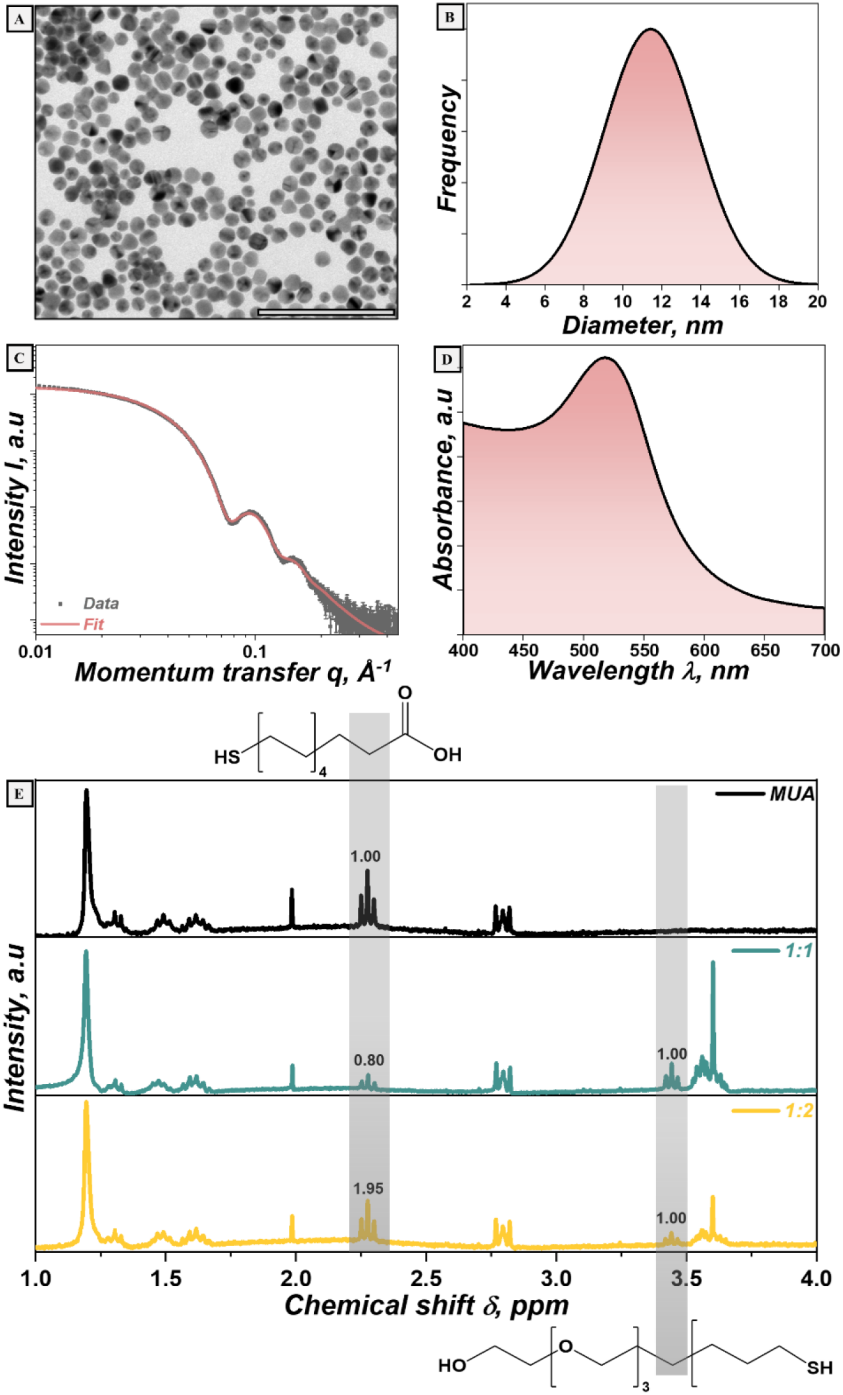
Gold nanocrystals (Au NCs) with mixed ligand layers used in this work. (A) TEM image (scale bar: 100 nm) showing the cores, (B) DLS indicating the distribution of hydrodynamic diameters, (C) SAXS scattering and model-based fit, and (D) UV–vis absorbance spectra of the synthesized 12 nm gold nanocrystals dispersed in water. (E) ^1^H NMR spectra of the gold nanocrystals coated with different molar ratios of TEG and MUA thiol ligands after removal of the Au cores: only MUA (in gray), equimolar ratio (in turquoise), and excess MUA (in yellow).

We replaced the citrate on the surface of Au NCs by ligand exchange with 11-mercaptoundecanoic acid (MUA), a negatively charged ligand, or combinations of MUA and triethylene glycol mono-11-mercaptoundecyl ether (TEG), a neutral ligand. Ligand combinations in solution have been reported to form mixed layers on Au NCs.^66^ Different TEG:MUA molar ratios were used in the reaction: pure MUA (in gray, Figure 1E), an equimolar mixture (in turquoise, Figure 1E), and an excess of MUA (in yellow, Figure 1E).

The resulting dispersions were washed and analyzed to assess the composition of the shells. Thiol-coated Au NCs were dissolved in aqua regia, diluted with D_2_O, and analyzed via ^1^H NMR. The molar ratios of the thiol-bound ligands were extracted by integrating the peak areas of the two triplets at 2.25 ppm (for MUA) and 3.40 ppm (for TEG) (Figure 1E). They were *n*_TEG_/*n*_MUA_ = 0/1 (hereafter 0), *n*_TEG_/*n*_MUA_ = 1/0.8 (hereafter 1.25), and *n*_TEG_/*n*_MUA_ = 1/1.95 (hereafter 0.51). The deviations from the ratios of added ligands during the ligand exchange reactions (Figure 1E) are expected due to differences in adsorption/desorption rates of the two ligand types.^66,67^ Reported ligand densities of Au NCs prepared in the same way were 4.88, 1.50, and 1.59 ligands/nm^2^ for *n*_TEG_/*n*_MUA_ = 0, *n*_TEG_/*n*_MUA_ = 0.51, and *n*_TEG_/*n*_MUA_ = 1.25, respectively.^66^ The zeta potentials of the Au NCs were measured using electrophoretic light scattering. The citrate-coated Au NCs displayed a negative zeta potential of −41.7 ± 1.17 mV, MUA-coated Au NCs had −50.7 ± 1.22 mV. Mixed shells of MUA and TEG had −52.0 ± 1.07 mV for *n*_TEG_/*n*_MUA_ = 1.25 and −60.8 ± 0.92 mV for *n*_TEG_/*n*_MUA_ = 0.51.

### 3.2 Parameters Affecting Colloidal Stability

We used UV–vis absorbance spectra to quantify the propensity of Au NCs to form agglomerates or assemblies in the presence of different salts, as listed in Table 1, along with their concentrations. The LSPR shifts of thiol-coated Au NCs (*n*_TEG_/*n*_MUA_ = 0, *n*_TEG_/*n*_MUA_ = 0.51, and *n*_TEG_/*n*_MUA_ = 1.25) were monitored as a function of the ionic strength of each salt, $I=\frac{1}{2}\sum c_{i}{z_{i}}^{2}$ , where *c*_*i*_ is the molar concentration and *z*_*i*_ is the valency of ion type *i*. A micropipetting robot provided precise timing such that all samples were prepared within 5 min, reducing age differences in the agglomerating mixtures. Aqueous salt solutions in a concentration range from 0.16 mM to 2000 mM were pipetted into defined positions on the well plate in approximately 40 min. Thiol-coated Au NCs were added to the aqueous solution of the salts within the last 5 min, left for 5 min, and then transferred to the UV–vis spectrometer. The readout of the 384-well plate was completed in 30 min, with each well being read in approximately 5 s. Agglomeration was detected from a redshift of the plasmonic absorption band of the Au NCs, λ_LPSR_ (i.e., the wavelength at maximum absorbance of the Au NCs). Such shifts are caused by changes in their local dielectric environment due to interparticle coupling.^68^

Figure 2A presents exemplary absorption spectra of Au NCs with *n*_TEG_/*n*_MUA_ = 0 and NaCl at various ionic strengths. For *I*_NaCl_ between 0.16 mM and 40 mM, λ_LPSR_ remained at ∼530 nm, with slight variations in absolute peak absorbance (Figure 2A). Au NCs with *n*_TEG_/*n*_MUA_ = 0.51 and *n*_TEG_/*n*_MUA_ = 1.25 showed slightly lower λ_LPSR_ at low *I*, indicating that the type of ligand shell influences the local dielectric environment of the metallic cores (Figure S2). Above 100 mM, λ_LPSR_ shifted to ∼580 nm, indicating interparticle coupling due to agglomeration. Asymmetric broadening of the SPR band, as compared to the spectra obtained prior to agglomeration, suggests increasing variations in the local dielectric environment, presumably due to heterogeneous agglomeration structures.^69^ Other salts showed similar trends, albeit with different *I* at which the shift in λ_LPSR_ occurred (Figure S3).

**Figure 2:**
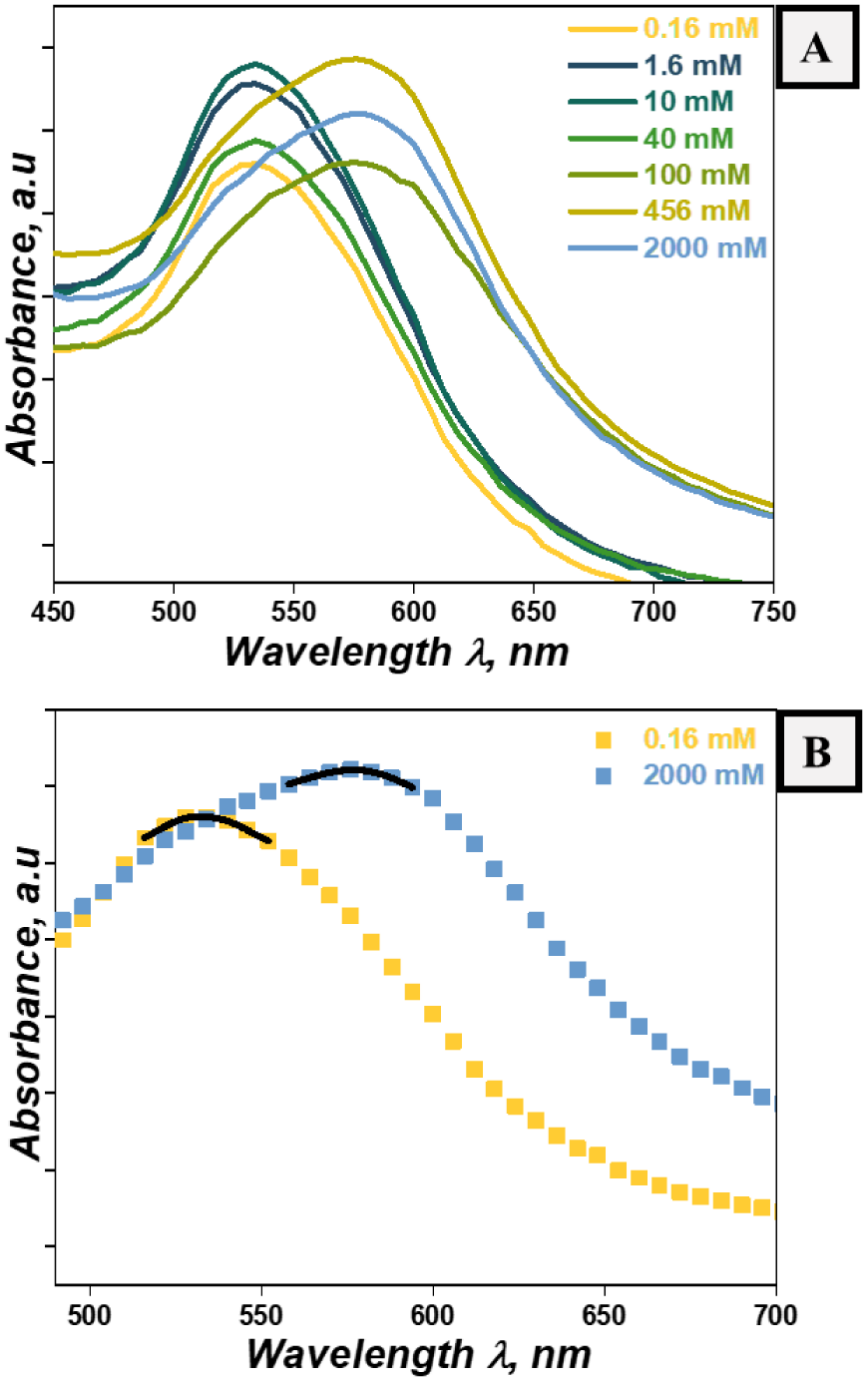
Plasmonic signatures of particle agglomeration: (A) Surface plasmon resonance peak of MUA-coated Au NCs at different ionic strengths of NaCl. (B) Determination of λ_LPSR_ from the absorbance spectra: The data in the vicinity of λ_LPSR_ were interpolated with cubic splines (black lines) to reliably find the local maximum used to detect agglomeration.

We quantified the colloidal stability using the value of λ_LSPR_. The resonance maximum λ_LPSR_ was determined by fitting cubic splines to the range around the plasmon peak (Figure 2B; see the experimental section for more details). For *n*_TEG_/*n*_MUA_ = 0, where Au NCs were negatively charged with a zeta potential of −50.7 ± 1.22 mV, λ_LSPR_ remained at around 530 nm for *I* < 10^–3^ M for most salts (Figure 3A), indicating well-dispersed Au NCs. Note the exception of AlCl_3_: it caused a strong shift already at the lowest measured *I*. Increasing to *I* > 1 mM increased λ_LSPR_ to up to 570–600 nm for all salt types. We interpreted a shift above the initial λ_LSPR_ (in the range of 525–530 nm, depending on *n*_TEG_/*n*_MUA_) as the onset of agglomeration and defined *I*_agglo_ as the critical ionic strength for the corresponding salt type (Figure 3A).

**Figure 3:**
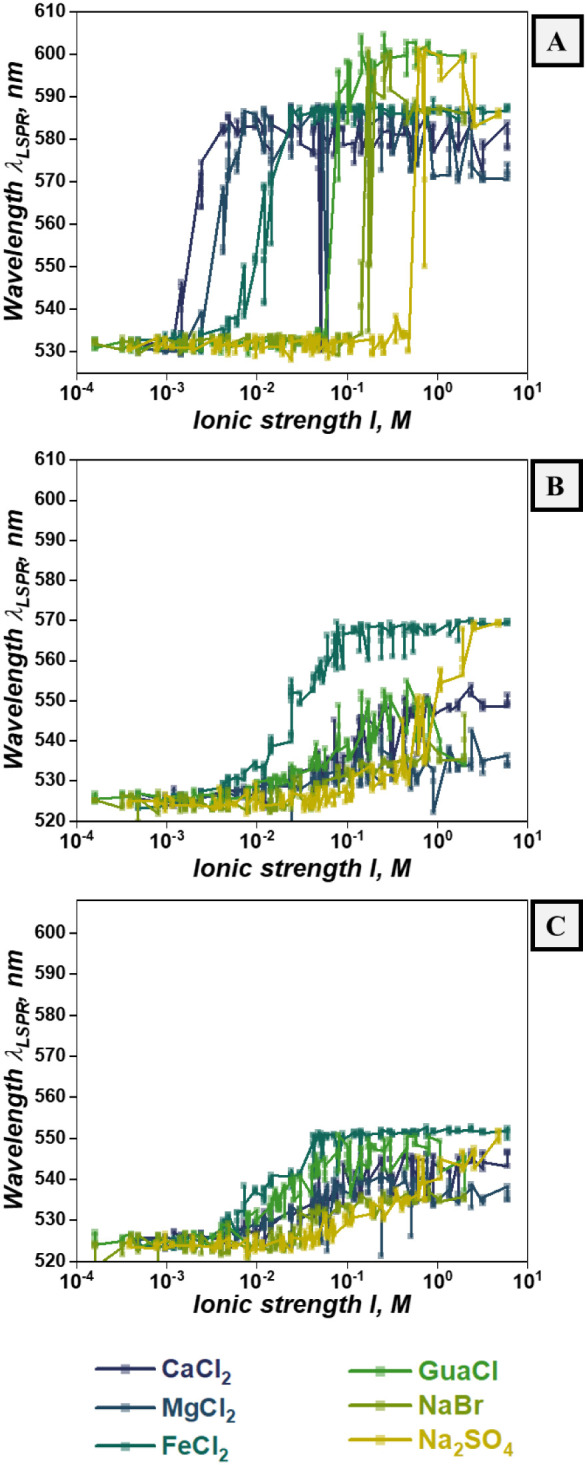
Surface plasmon resonances λ_LSPR_ of Au NC dispersions with ligand ratios in electrolytes: (A) *n*_TEG_/*n*_MUA_ = 0, (B) *n*_TEG_/*n*_MUA_ = 0.51, and (C) *n*_TEG_/*n*_MUA_ = 1.25 as a function of the ionic strengths of the selected salts.

The lowest *I*_agglo_ was found for AlCl_3_ at below 0.96 mM, followed by CaCl_2_ with 1.1 mM, while Na_2_SO_4_ had the highest *I*_agglo_ at 0.48 M (Figure 3A). The agglomeration efficacy of the cations (with Cl^–^ as an anion) followed the trend Al^3+^ > Ca^2+^ > Mg^2+^ ≈ Sr^2+^ > Fe^2+^ > Na^+^ ≈ Gua^+^ > NH_4_^+^ > K^+^. The anions (with Na^+^ as a cation) followed H_2_PO_4_^–^ > Cl^–^ > I^–^ > CO_3_^2–^ ≈ F^–^ ≈ Br^–^ > HPO_4_^2–^ > SO_4_^2–^ > S_2_O_3_^2–^. Divalent cations had stronger agglomeration efficacy than monovalent cations, while monovalent anions had stronger agglomeration efficacy than divalent anions. The agglomeration efficacy did not follow Hofmeister’s series,^70^ nor the order reported for Au NCs coated with citrates.^35^

The colloidal stability of MUA-coated gold colloids followed the predictions of classical DLVO theory.^71^ Increasing ionic strengths reduced the Debye screening length and colloidal stability. Multivalent cations had a stronger agglomeration efficacy than monovalent cations, as predicted by DLVO’s Schulze–Hardy rule.^72,73^ The opposite trend (“inverse Schulze–Hardy rule”) for anions has been shown to be consistent with DLVO too: as their valency increases, electrostatic repulsion between ions and the charged particle surface becomes stronger, hindering adsorption.^74^ Our observations for the agglomeration efficacies of anions followed this rule without exception.

Nanoparticles with mixed ligand shells, *n*_TEG_/*n*_MUA_ = 0.51 (Figure 3B) and *n*_TEG_/*n*_MUA_ = 1.25 (Figure 3C), did not always follow trends predicted by DLVO. These particles generally required higher *I* to redshift λ_LSPR_ as compared to *n*_TEG_/*n*_MUA_ = 0 (Figure 3A), indicating increased colloidal stabilities. The maximum λ_LSPR_ shifts were lower for *n*_TEG_/*n*_MUA_ = 0.51 and *n*_TEG_/*n*_MUA_ = 1.25 than for *n*_TEG_/*n*_MUA_ = 0. This value was within 530 to 570 nm, depending on the type of salt, suggesting weaker coupling between Au NCs with mixed shells.^69^ We do not know at present whether this is due to larger spacings or lower agglomerate densities that reduce couplings.

Zeta potential measurements indicated that the charges of Au NCs became more negative with increasing TEG fraction: *n*_TEG_/*n*_MUA_ = 0.51 had −52.0 ± 1.07 mV, *n*_TEG_/*n*_MUA_ = 1.25 had −60.8 ± 0.92 mV, respectively. This is surprising given the negative charge of MUA. It may be caused by the molecular ligand shell structure, where densely packed MUA is less likely to be deprotonated at a given pH and pI than more dilute MUA in a TEG environment. Irrespective of the underlying mechanism, we found that particles with larger absolute zeta potentials agglomerated at larger *I*, as expected. Note that charge may not be the only contribution to stability here. TEG is more hydrophilic than MUA because of its ether group, so the hydrophobic interaction between particles likely reduced with increasing ligand ratio. It is conceivable that the intrinsic entropic nature of mixed ligand shells promotes disorder of the shell, thereby stabilizing the dispersion as well.^75^ We assume that the acidity of MUA is sufficiently large to minimize the effects of different pH in the unbuffered salt solutions.

The order of cations’ and anions’ agglomeration efficacies for *n*_TEG_/*n*_MUA_ = 0.51 was Al^3+^ > NH_4_^+^ > Fe^2+^ > Ca^2+^ ≈ Mg^2+^ ≈ Sr^2+^ ≈ Gua^+^ ≈ K^+^ > Na^+^ with Cl^–^, and H_2_PO_4_^–^ > F^–^ ≈ Br^–^ > SO_4_^2–^ > Cl^–^ ≈ I^–^ ≈ S_2_O_3_^2–^ > CO_3_^2–^ > HPO_4_^2–^ with Na^+^. The trends loosely followed the Schulze–Hardy rule for cations and the inverse Schulze–Hardy rule for anions, with exceptions: the monovalent NH_4_^+^ had the second strongest agglomeration efficacy, and the divalent SO_4_^2–^ had a stronger agglomeration efficacy than Cl^–^ and I^–^. Particles with *n*_TEG_/*n*_MUA_ = 1.25 agglomerated in wide ranges of *I*.

In summary, agglomeration efficiencies for ions with the same valencies differed for Au NCs with *n*_TEG_/*n*_MUA_ = 0 and *n*_TEG_/*n*_MUA_ = 0.51. The DLVO theory does not account for specific ion effects and cannot explain these differences. It is likely that the specific ion effects occur when the electrostatic screening length becomes small, and intrinsic ion properties such as size, shape, and polarizability dominate the local ion-ligand interaction.^76^ In the following, we present a machine learning approach to determine the extent to which certain specific ion properties contribute to the colloidal stability of MUA/TEG-coated Au NCs.

### 3.3 Machine Learning

Several intrinsic properties of an ion can substantially affect the colloidal stability of ligand-coated nanocrystals.^77^ First, electrostatic interactions, linked to the ion’s valency and radius, are involved in charge screening and dominate the interaction of the ions with their solvent environment.^78^ Second, the short-range vdW forces of ions with solvent molecules and solutes are determined by the ion’s polarizability,^31,79–81^ which may additionally lead to cavitation forces.^82,83^

In the following, we use an agnostic “black box” approach to analyze the sensitivity of λ_LSPR_ to different salts. A Random Forest Regression (RFR) model^84^ correlated intrinsic ion properties and system variables with λ_LSPR_. We chose descriptors that have been reported to affect the agglomeration efficacy of ions^34–38^ and included the cation (*R* ^+^) and anion (*R* ^–^) radii, cation (α^+^) and anion (α^–^) polarizabilities, and cation (*q* ^*+*^) and anion (*q* ^–^) valencies. The ionic strengths (*I*) of the salts and the ligand ratio (*n*_TEG_/*n*_MUA_) were included as system variables as well (see Table 1 for a full list of ion radii, valencies, and polarizabilities of the explored salts).

First, the performance metrics of the RFR model were determined by repeated train-test splitting. Figure 4 shows the predicted λ_LSPR_ in dependence on the measured ones for both the train and test sets of one 80/20 train-test split, visualizing the model’ predictive performance. No obvious difference in prediction accuracy of the model is discernible, and outliers were present in both cases to a similar extent, both pointing to a good model generalization quality. Averaging over 100 different train and test sets, an average mean coefficient of determination (*R* ^2^) of 0.959 with a standard deviation of 0.020 was calculated, indicating excellent predictive performance and reproducibility of the built model. Calculations of the MSE of the train and test sets separately yielded a train-MSE of 6.811 ± 0.301 nm² and a test-MSE of 12.458 ± 1.717 nm^2^. The difference between both may indicate some degree of overfitting.^85^ However, reducing the tree depth in the RFR model increases the training error more than the test error, indicating that the simplified model loses important structure rather than meaningfully improving generalization. Therefore, the current model configuration is well-balanced for the given dataset.

**Figure 4:**
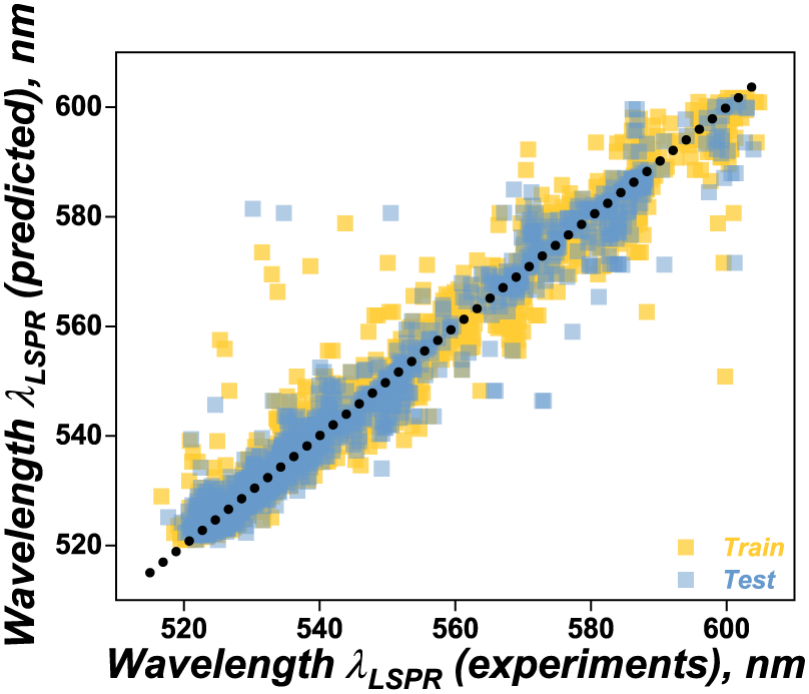
Plasmon resonance wavelength λ_LSPR_ as predicted by the RFR model in dependence on the measured λ_LSPR_.

Classical DLVO theory models the critical coagulation concentration of different electrolytes and particle properties as a function of their variables. We can use the RFR model to recover similar relations for the effect of *n*_TEG_/*n*_MUA_ and the intrinsic ion properties of *I* on λ_LSPR_. Our analysis shows how sensitively λ_LSPR_ depends on all descriptors and allows us to systematically predict the effect of each parameter on λ_LSPR_ (Figure 5). Note the similarity to classical DLVO, where partial derivatives are commonly used to predict the role of a single parameter in agglomeration. In the following, we use the RFR results to discuss the onset of agglomeration, *I*_agglo_, and the structure of the agglomerates via λ_LSPR_ at the largest measured *I* separately.

**Figure 5:**
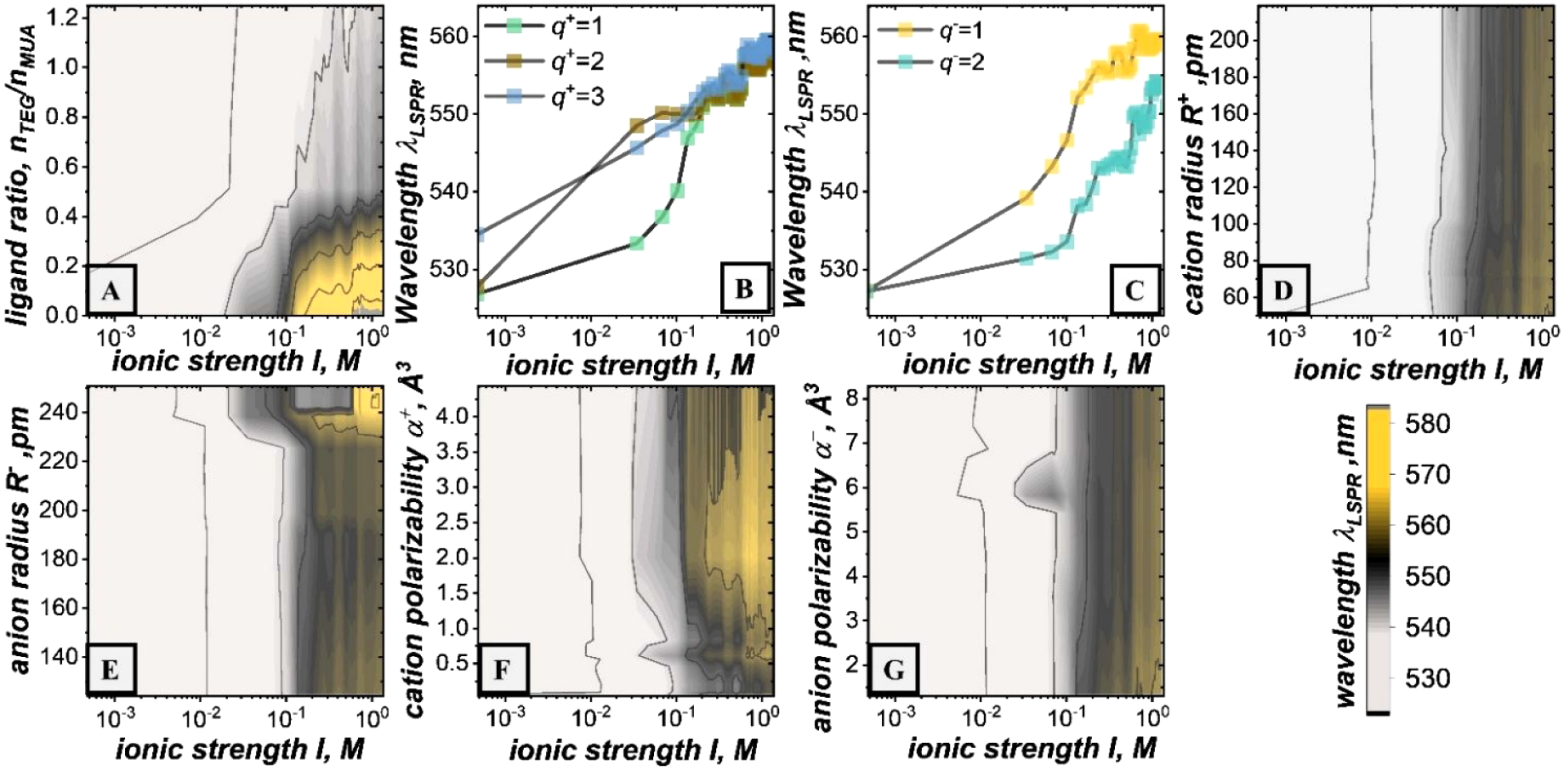
Shifts of plasmonic resonance maxima by salt solutions of different ionic strengths *I* as a function of (A) molar ratio *n*_TEG_/*n*_MUA_, (B) cation valency *q* ^+^, (C) anion valency *q* ^–^, (D) cation radius *R* ^+^, (E) anion radius *R* ^+^, (F) cation polarizability α^+^, and (G) anion polarizability α^+^. In all graphs, the black lines are contour lines.

Particles with *n*_TEG_/*n*_MUA_ = 0 had the lowest *I*_agglo_ (Figure 5A), consistent with the direct analysis from Figure 3A. Higher *n*_TEG_/*n*_MUA_, i.e., an increased concentration of TEG within the shell, enhanced colloidal stability in a highly nonlinear fashion because of the increased surface charge and hydrophilicity, as discussed above.

The ion valencies *q* ^+^ and *q* ^–^ (Figure 5B,C) affected agglomeration, as discussed above, too. Cations and anions followed the Schulze–Hardy and inverse Schulze–Hardy rules, respectively: increasing *q* ^+^ increased λ_LSPR_ at low *I*, in the range where Au NCs agglomerated, pointing to a stronger agglomeration efficacy. The trend was reversed for *q* ^–^.

*I*_agglo_ decreased with increasing *R* ^–^ (Figure 5E) and with decreasing *R* ^+^ (Figure 5D), showing opposite trends for anions and cations. Ions affect the local hydrogen-bonding structure of water, an effect that is not considered in DLVO theory. Anions mainly interact with water through electrostatic interactions, while for cations, vdW forces compete more strongly with Coulombic interactions.^31,32^ The electrostatic interaction between ions and the local water scales with the ions’ radial charge density, $q^{-}/\sqrt[{3}]{\left\langle R^{-3}\right\rangle }$ . Large ions are typically weakly hydrated (they enhance the solubility of hydrophobic substances) because they have a relatively low charge density. The negative correlation between *I*_agglo_ and *R* ^–^ suggests that anions engage in electrostatic interactions different from those with their local water structure to affect *I*_agglo_. Conversely, cations do affect the colloidal stability of Au NCs through the local water structure. Additionally, as cations may also interact with the ligand shell directly, those with a small *R* ^+^ can enter the ligand shell more easily, promoting agglomeration.

Finally, α^–^ only marginally affected *I*_agglo_, while a large α^+^ increased it (Figure 5F,G). This is consistent with a picture where the anions are mainly involved in electrostatic interactions with their water environment, while cations act through vdW interactions, too.^86^

The structure of the agglomerates strongly depended on *n*_TEG_/*n*_MUA_ (Figure 5A). At the highest measured *I* of 2 M, when Au NCs with all ligand ratios had agglomerated, λ_LSPR_ was highest for small *n*_TEG_/*n*_MUA_, i.e., with a small fraction of TEG. λ_LSPR_ depends sensitively on interparticle distances, *s*, with λ_LSPR_ increasing for decreasing *s*.^68^ Apparently, a smaller fraction of the large TEG within the shell enables Au NCs to come closer, making the agglomerates denser.

The resonance λ_LSPR_ at *I* = 2 M did not depend much on *q* ^+^, suggesting that *q* ^+^ did not affect the agglomerate structure. In contrast, low *q* ^–^ increased λ_LSPR_ at *I* = 2 M, pointing to smaller interparticle distances within agglomerates. Furthermore, λ_LSPR_ at *I* = 2 M increased with increasing *R* ^–^. Both observations point to a negative correlation between λ_LSPR_ at *I* = 2 M and the anion radial charge density. Anions and cations apparently caused agglomeration by different mechanisms.

Smaller *R* ^+^ led to marginally larger λ_LSPR_ at *I* = 2 M. Smaller cations can enter the ligand shell more easily, thereby reducing the screening length, which leads to smaller interparticle distances. The increase of λ_LSPR_ at *I* = 2 M with α^+^ (Figure 5F) is consistent with this interpretation: cation-ligand vdW attraction disrupts the hydration layer or binds adjacent Au NCs, both leading to agglomeration of Au NCs. The polarizability of anions α^–^ did not affect λ_LSPR_ significantly (Figure 5G). Anions are not attracted by equally charged ligands, and their vdW attraction does not play a large role.

It is interesting to quantify the relative relevance of all descriptors (properties of the ions, concentrations, and shell compositions) for the prediction accuracy of the model, similar to interpreting partial derivatives of an analytical expression. The permutation feature importances of all descriptors, a standard measure for the decrease in model accuracy when the values of each descriptor are permuted,^84^ were determined using *R* ^2^ as the performance metric and are shown in Figure 6. Both *n*_TEG_/*n*_MUA_ and *I* dominated the agglomeration efficacy over the intrinsic ion properties, namely their *q*, *R*, and α. This agrees with the dependence of λ_LSPR_ on *I* (Figure 3), which dominated for each salt type.

**Figure 6:**
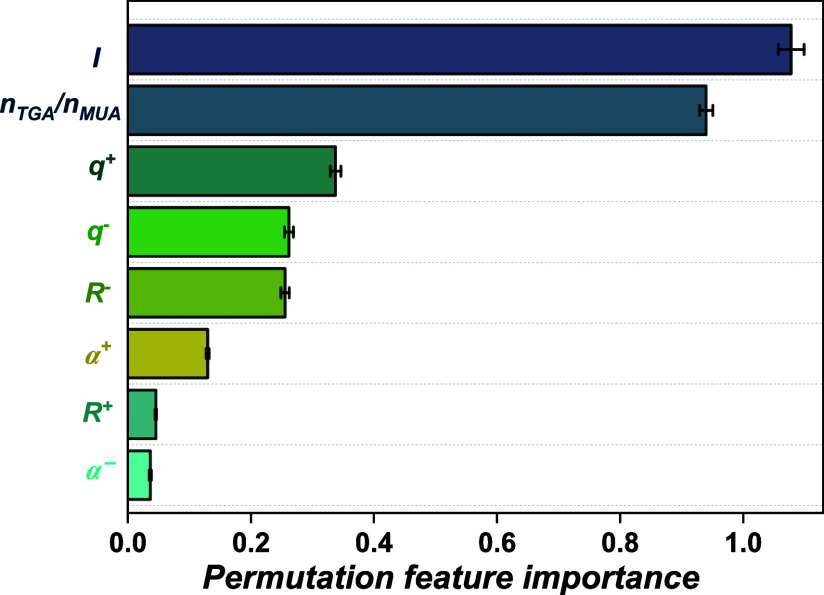
Ranking of descriptor importance to the colloidal stability of Au NCs based on their permutation feature importance.

The ligand ratio determined the width of the range of *I* at which agglomeration takes place and the maximum value of λ_LSPR_. This value is related to the agglomerate structure, as described above. The type of salt merely shifted the ionic strength at which agglomeration of Au NCs sets in.

The ion valencies *q* ^+^ and *q* ^–^ were the intrinsic ion properties that most strongly affected colloidal stability. This is consistent with the direct analysis above: the agglomeration efficacies of many salt-particle combinations followed the Schulze–Hardy rule for cations and the inverse Schulze–Hardy rule for anions. The effect of the type of ion with the same co- or counterion valency was only secondary. The ranking of valencies is consistent with our observation that α^+^ and *R* ^–^ affected the agglomeration efficacy of Au NCs more than *R* ^+^ and α^–^ (Figure 6): as anions interact mainly through electrostatic interactions with the local water environment,^31,32^ *R* ^–^ strongly affects it. On the other hand, the significant contribution of vdW interaction of cations to the local water structure leads to a high relative importance of α^+^.

Lastly, direct vdW interactions between ions and the charged Au NCs are determined by ion polarizabilities, too. Our Au NCs were negatively charged and more affected by vdW interactions with cations than anions, ranking α^+^ above α^–^. Beyond screening the particles’ surface charges, cations may also act as “ionic glue” that binds Au NCs within agglomerates.^33^

Note that RFR ranking was done without prior classification into “agglomerating”, “stable”, or “partially agglomerating” dispersions. Thus, the importance of the different parameters shown in Figure 6 does not rank the agglomeration efficacy as in the case of DLVO theory, where critical ion concentrations are compared. Instead, the RFR ranking indicates which parameter has the strongest effect on the overall plasmonic shift. This combines the degree of agglomeration (fraction of agglomerated particles), spacing between particles in the agglomerate, and the density of the agglomerate. From a practical standpoint, the ranking is particularly useful to determine how a maximal optical effect can be achieved. From a fundamental standpoint, it raises questions about agglomerate structure that can only be answered through a structural analysis of the agglomerates.

## 4 Conclusions

In this study, we assessed the stability of aqueous dispersions of neutral or negatively charged gold nanocrystals (Au NCs) in the presence of a broad range of salts and salt concentrations using UV–vis absorption spectroscopy. The change in surface plasmon resonance was used to identify agglomeration. An automated high-throughput approach was chosen to enable the testing of a wide range of ionic strengths with narrow concentration steps and to find critical concentrations of agglomeration or concentration ranges of agglomeration. This enabled the testing of on the order of 10000 different combinations of particles, salts, and concentrations to study subtle and strong deviations from classical colloidal theory and to delineate areas where nanoparticle agglomeration deviated from classical colloidal systems.

The results were analyzed using both classical DLVO interpretation and an algorithmic approach based on random forest regression. This machine-learning algorithm can manage the large parameter space and disentangle the relative importance of electrostatic and vdW interactions in nanoparticle agglomeration. The results of both analyses overlapped to a large degree, indicating that DLVO is applicable if the interparticle distance is sufficiently large.

The influence of ion valencies on the electrostatic screening of the negatively charged Au NC surfaces was consistent with DLVO theory and followed the Schulze–Hardy rule for cations and the inverse Schulze–Hardy rule for anions. Beyond these classical electrostatic effects, contributions not captured by the DLVO theory were identified. Anions influenced colloidal stability not only through direct electrostatic interactions with the Au NC surfaces but also through their effect on the local water structure.^87,88^ In contrast, cations significantly affected the local water structure through vdW interactions as well and additionally interacted directly with the charged surface ligands.^87,88^

In future work, other features of the absorbance spectra will be evaluated to extend this work beyond the differentiation between stable and agglomerating nanoparticle dispersions. We expect that it will be possible to address agglomerate size, size distributions, and agglomerate packing densities by analyzing optical signatures and comparing them with X-ray scattering results. This requires high-throughput small-angle X-ray scattering approaches that are currently being developed.

## Supplementary Material



## Notes

High-resolution transmission electron images of gold nanocrystals; performance metrics linked to the learning algorithms (PDF)
