# Supplementary material for: Agglomeration Efficacies of Simple Salts on Charged Gold Nanocrystals with Mixed Ligand Shells: A High-Throughput Study
Source: ACS Mater Au. 2026 May 9;6(4):860–71. doi: 10.1021/acsmaterialsau.6c00051 (PMC13352275; doi:10.1021/acsmaterialsau.6c00051)
Supplement: Supplementary file 1 [file mg6c00051_si_001.pdf]

Supporting Information for: Agglomeration efficacies of simple salts on charged gold nanocrystals with mixed ligand shells: a high-throughput study

Albenc Nexha,[1,§] Bart-Jan Niebuur[1,§] Simon Blum,[1] and Tobias Kraus[1,2*]

[1] *INM-Leibniz Institute for New Materials, Campus D2 2, 66123 Saarbrücken, Germany*
[2] *Colloid and Interface Chemistry, Saarland University, 66123 Saarbrücken, Germany*
*tobias.kraus@leibniz-inm.de
[§]These authors contributed equally to this work

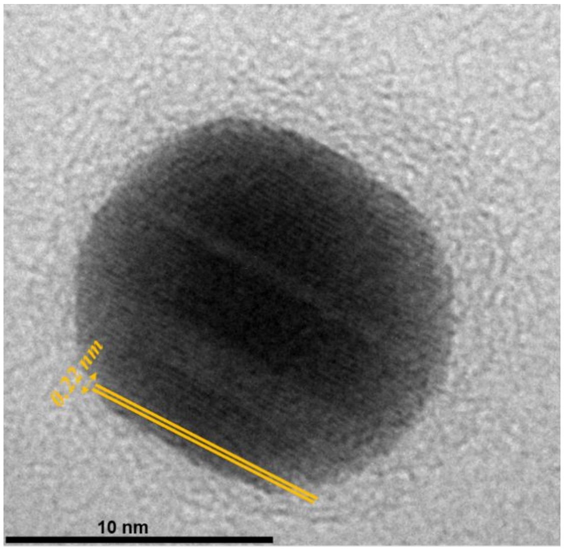

Figure S1. High magnification TEM image of the citrate coated gold nanoparticles synthesized via the seeded growth protocol.

Table S1. Performance metrics coefficient of determination, R^2, root mean square error (RMSE) and mean absolute error (MAE) of 4 ensemble machine learning algorithms as determined using an 80/20 train-test split of the recorded data.

**Algorithm**	R^2	**RMSE**	**MAE**
Gradient Boosting Regression	0.9619	3.8071	1.9627
XGBoost Regression	0.9622	3.7900	1.9516
CatBoost Regression	0.9639	3.7057	1.9388
Random Forrest Regression	0.9654	3.6286	1.9506

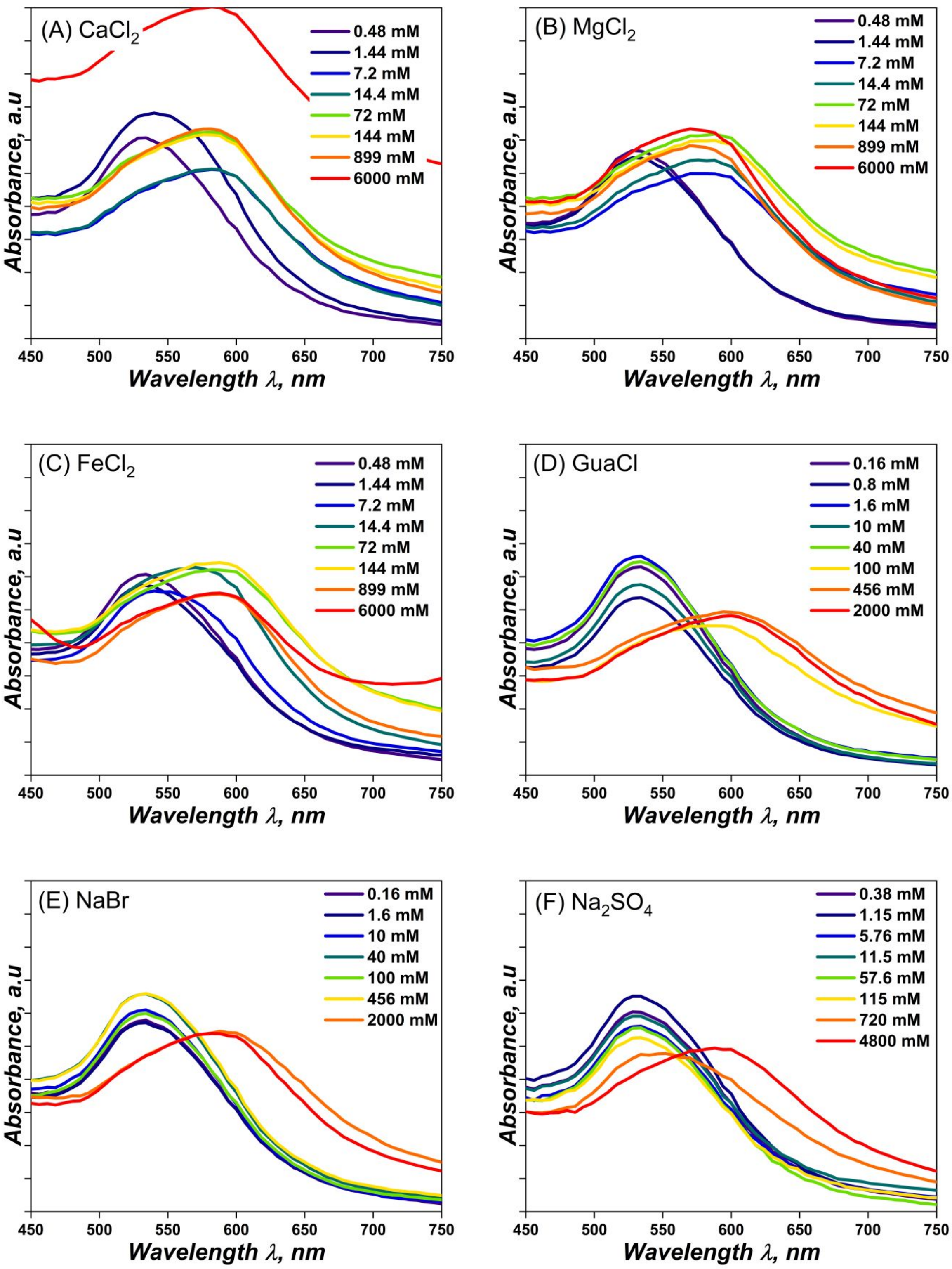

Figure S2. Surface plasmon resonance peak of MUA coated Au NCs in the presence of (A) $CaCl_2$, (B) $MgCl_2$, (C) $FeCl_2$, (D) GuaCl, (E) NaBr and (F) Na_2SO_4 at ionic strengths as indicated in the graphs.

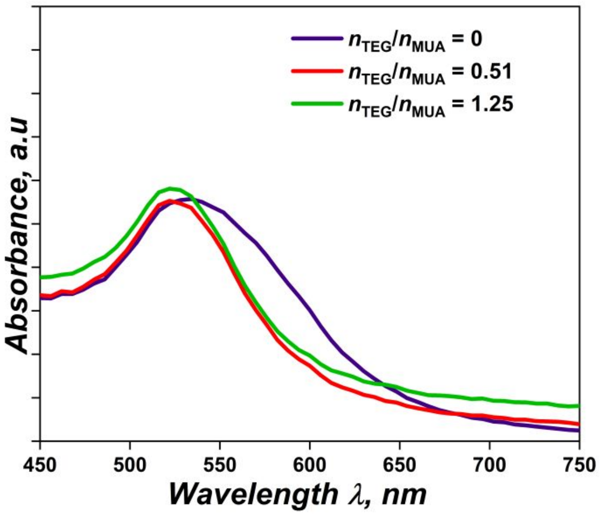

Figure S3. Surface plasmon resonance peak of Au NCs in the presence of NaCl at an ionic strength of 0.16 mM and ligand ratios n_{TEG}/n_{MUA} as indicated in the graph.
